# Portal Vein Thrombosis in COVID-19: An Underdiagnosed Disease?

**DOI:** 10.3390/jcm13185599

**Published:** 2024-09-21

**Authors:** Ismael El Hajra, Elba Llop, Santiago Blanco, Christie Perelló, Carlos Fernández-Carrillo, José Luis Calleja

**Affiliations:** 1 Department of Gastroenterology and Hepatology, Hospital Universitario Puerta de Hierro Majadahonda, 28222 Madrid, Spain; santiblancorey@gmail.com (S.B.); draperello@gmail.com (C.P.); cfernandezc@idiphim.org (C.F.-C.); joseluis.calleja@uam.es (J.L.C.); 2 Instituto de Investigación Sanitaria Puerta Hierro-Segovia Arana (IDIPHISA) Majadahonda, 28222 Madrid, Spain; 3 Centro de Investigación Biomédica en Red (CIBEREHD), 28029 Madrid, Spain

**Keywords:** portal vein thrombosis, COVID-19, thromboembolism

## Abstract

**Background:** Multiple studies have linked COVID-19 to a higher incidence of thromboembolic disorders. However, the association of COVID-19 with other potentially life-threatening complications, such as splanchnic vein thrombosis, is less well understood. This study aims to assess the prevalence, patient characteristics, clinical presentation, and outcomes of patients with portal vein thrombosis (PVT) and COVID-19. **Methods:** This was a retrospective observational study. From all positive patients for a reverse-transcription polymerase chain reaction (RT-PCR) swab test from March 2020 to June 2020, we included those who were older than 18 years, had received abdominal contrast-enhanced computed tomography (CT) in the 6 months following the positive RT-PCR swab, and had no previously known splanchnic vein thrombosis. **Results:** A total of 60 patients with abdominal CT were selected from all those positive for SARS-CoV-2 (*n* = 2987). The prevalence of PVT was 3/60 (5%). The mean age was 66.1 ± 16.5 years and 51.7% were male. In two of the three patients, there was no underlying pathology as a risk factor for PVT and one of them presented cirrhosis. The number of days from the start of COVID-19 symptoms until the PVT diagnosis were 21, 12, and 10 days. Anticoagulation treatment achieved recanalization in 100% of cases. During a mean follow-up of 803 days, none of the patients experienced long-term complications. **Conclusions:** Portal vein thrombosis is uncommon, and its incidence may be higher in COVID-19 patients. A greater understanding of the features of this disease in the context of COVID-19 could aid towards its diagnosis and allow for early detection and management.

## 1. Introduction

After the first case of COVID-19 was reported in December 2019, the number of cases increased rapidly and spread globally. Although COVID-19 is a pathology that mainly affects the lower respiratory tract, other extra-respiratory manifestations have been described, such as cardiovascular, gastrointestinal, or thromboembolic conditions, making it a multisystem disease. Multiple studies have pointed out the great prothrombotic capacity of SARS-CoV-2 given the greater incidence of deep vein thrombosis (DVT) and pulmonary thromboembolism (PE) in patients without risk factors than in other respiratory infections [1,2,3].

The pathogenesis of the COVID-19-related hypercoagulable state and thrombosis is not yet fully understood. The main mechanisms involved include endothelial injury and prothrombotic changes in coagulation. There is evidence of a direct invasion of endothelial cells by SARS-CoV-2, which leads to endothelial damage. Moreover, the presence of the virus causes an activation of the host defense mechanism that induces a hyperinflammatory response to infection where a cytokine storm or cytokine release syndrome can contribute to additional tissue damage and generate a prothrombotic state through multiple mechanisms, such as the activation of neutrophils and monocytes, which increases the risk of micro- and macrovascular thrombosis [4,5].

Cases of portal vein thrombosis (PVT) in the absence of liver cirrhosis, malignancy, or acute abdominal inflammation are rare, and acute PVT shows a wide spectrum of clinical presentations ranging from asymptomatic cases to cases where thrombosis within the portal system can rapidly expand and the clot may progress into the mesenteric venous system, leading to severe intestinal ischemia and infarction [6].

An analysis of liver histopathology in a large autopsy series of COVID-19 patients showed a high prevalence of fibrosis and thrombosis of portal and sinusoidal vessels accompanied by massive pericyte activation [7,8]. When these cells are activated, they express myofibroblast markers that produce wall fibrosis with the perturbation of microvascular perfusion owing to vasoconstriction and occlusion. The hepatic microvascular alterations are associated with intrapulmonary vascular dilations which exacerbate the respiratory failure of COVID-19 patients [9]. Therefore, the phenomena of thrombosis and microthrombosis in the splenoportal axis may be related to more severe COVID-19 infection with progressively worsening respiratory failure.

Furthermore, another important point is that portal vein thrombosis in patients with SARS-CoV-2 infection has been associated with a worse prognosis with a higher risk of intestinal infarction requiring resection [10]. These findings suggest that the severity of thrombosis in the splanchnic territory could be different during SARS-CoV-2 infection and with it, the prognosis.

Although the incidence of deep vein thrombosis and pulmonary embolism in patients with COVID-19 is well documented, the clinical information available on thrombosis in the splenoportal axis is limited to isolated case reports, small case series, or autopsy data. The objective of this study was to determine the prevalence, clinical symptoms, patient characteristics, and outcomes of patients with portal vein thrombosis in a cohort of COVID-19 patients.

## 2. Materials and Methods

This was a retrospective observational study performed in a tertiary care hospital. From all consecutive eligible patients who were positive for a reverse-transcription polymerase chain reaction (RT-PCR) swab in our hospital from March 2020 until June 2020, patients were included in the study according to the following inclusion criteria: age > 18 years and abdominal contrast-enhanced computed tomography (CT) in the 6 months following the positive RT-PCR swab. Patients with previously known splanchnic vein thrombosis were excluded. Abdominal CT was performed upon indication by the treating physician and PVT was diagnosed based on the CT scan.

Demographic, hospital admission, clinical, radiological, and laboratory data obtained upon arrival at the emergency department or on admission, as well as during follow-up, were collected from electronic medical records. Respiratory severity was defined based on the Brescia scale as follows: 0 = non-oxygen therapy requirements; 1–3 = oxygen therapy requirements or non-invasive ventilation; and >3 = invasive ventilatory support. There was a hospital protocol in place during the initial phase of the outbreak (3 March to 30 March) in which COVID-19 was considered a disease with a standard risk of thrombosis and patients received 40 mg of enoxaparin subcutaneously (prophylactic dose). From April onwards, patients who required admission received 1 mg/kg of enoxaparin subcutaneously twice daily (therapeutic dose).

Tests for thrombophilia included testing for the factor-V-Leiden mutation, the G20210A prothrombin gene mutation, protein C and S deficiency, antithrombin deficiency, the JAK 2 mutation, and the exclusion of polycythemia vera, essential thrombocythemia and primary myelofibrosis, and antiphospholipid syndrome, as well as testing for autoimmune disease and vasculitis.

Follow-up examinations to assess recanalization were performed at 6 and 12 months with CT scans. The minimum follow-up of patients was two years, during which clinical and radiological data were collected, with all patients having an abdominal imaging test 6 months after discontinuing anticoagulation if recanalization was achieved.

Continuous variables are shown as the mean and standard deviation (SD) or as the median and interquartile range (IQR) depending on whether or not normality can be assumed (which was evaluated using the Shapiro–Wilk test). Categorical variables are presented as absolute and relative frequencies. Quantitative variables were compared using the Mann–Whiney test. A comparison of qualitative variables was performed using the Fisher exact test as a replacement for the chi-square test, since the expected frequency of one or more cells was less than 5. All tests are two-sided, and *p* < 0.05 was considered significant. An estimate of the cumulative incidence was made taking into account the number of new events and the total number of individuals at risk, considering those with a positive RT-PCR within a 6-month interval. The study complies with the Declaration of Helsinki.

## 3. Results

### 3.1. Patient Selection and Indications for Abdominal CT

**Figure 1:**
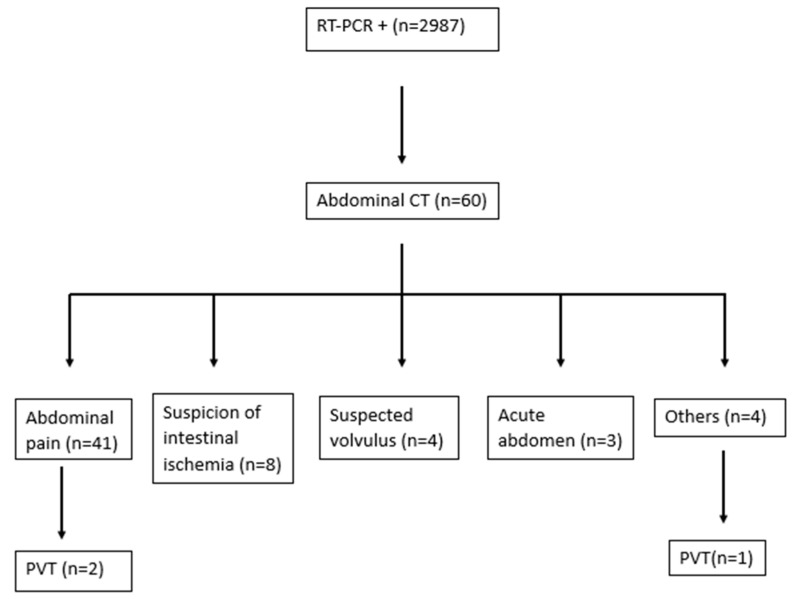
Flowchart of the study.

A total of 2987 patients were positive for RT-PCR, of which 60 received an abdominal CT scan and were included in the analysis (mean age of 66.1 ± 16.5 years and 51.7% were male). Abdominal CT was performed for the following conditions: abdominal pain (68.3%), suspicion of intestinal ischemia (13.3%), suspected volvulus (6.7%), acute abdomen (5%), and others (6.7%). Among the remaining reasons in other conditions for which the CT scan was performed were the monitoring of oncological disease in one case, suspected oncological disease in one case, data of intestinal obstruction in one case, and ascites in one case with suspicion of portal thrombosis in a performed Doppler ultrasound. A flowchart of the study is provided in Figure 1.

### 3.2. Prevalence of Portal Vein Thrombosis and Other Thromboembolic Events

The prevalence of PVT was three cases (5%), corresponding to an approximate 6-month cumulative incidence of one case per 1000 patients with COVID-19. Another nine thromboembolic phenomena were also observed, comprising six cases of pulmonary embolism and three of deep vein thrombosis.

### 3.3. Baseline Characteristics of Patients with Portal Vein Thrombosis

Of the patients with PVT, one patient had cirrhosis, while the other two did not present any liver disease. There was no history of thrombophilia or malignancy in any of the patients. Moreover, thrombophilia tests (including testing for the factor-V-Leiden mutation, the G20210A prothrombin gene mutation, the JAK 2 mutation, protein C and S deficiency, and antithrombin deficiency) were carried out after the diagnosis of PVT and were negative.

The mean age of patients with PVT was 48.33 ± 14.8 years and three (100%) were males vs. 67 ± 16.19 years (*p* = 0.056) and 28 (49.1%) males (*p* = 0.131) in the non-PVT group.

The most prevalent comorbidities in patients with PVT were hypertension (*n* = 2), diabetes (*n* = 1), and cardiovascular disease (*n* = 1). Nevertheless, the comorbidities did not differ significantly between the non-thrombotic and the thrombotic groups.

### 3.4. Clinical Characteristics and Outcomes

The presenting symptoms of PVT were abdominal pain in the two cases with no cirrhosis and ascites in the patient with cirrhosis. The number of days from the start of COVID-19 symptoms until the PVT diagnosis were 21, 12, and 10 days, respectively. 

No differences were found in the severity of COVID-19 in terms of the Brescia scale (67.7% in PVT vs. 40.4% in non-PVT; *p* = 0.565 in Brescia scale 1–3 and 0% in PVT vs. 3.5% in non-PVT, *p* = 1 in Brescia scale ≥ 4), extent of lung consolidations in chest X-rays (67.7% in PVT vs. 42.1% in non-PVT; *p* = 0.574 in 25–50% of lung consolidation and 0% in PVT vs. 24.6% in non-PVT; *p* = 1 in >50% of lung consolidation), need for ICU admission (0% in PVT vs. 15.8% in non-PVT; *p* = 1), or death (0% in PVT vs. 17.9% in non-PVT; *p* = 1).

In the case of patients with PVT, two of them received low-molecular-weight heparin (LMWH) at a therapeutic dose and one of them at a prophylactic dose in the first 24 h after admission.

On the abdominal CT, the spleen size was 8 and 9 cm in patients without cirrhosis and 14.5 cm in the patient with cirrhosis. The only patient who had a previous Doppler ultrasound was the patient with cirrhosis, whose spleen size was 14 cm, and the portal vein size was 11 mm. In this last patient, the liver stiffness was 24.5 kPa, having previously been 20 kPa. In the other two patients, liver stiffness was 4.7 and 5.4 kPa. Neither of them had splenic stiffness measurements.

All patients had a resolution of clinical symptoms and no short-term complications. Anticoagulation therapy was administered to all patients after diagnosis, and recanalization was achieved in 100% of cases at 6 months.

No differences in laboratory parameters (C-reactive protein, serum ferritin, LDH, CK, liver function tests, platelets, neutrophil-to-lymphocyte ratio, prothrombin time, fibrinogen, or D-dimer) were observed between patients with and without SARS-CoV-2 infection. The complete characteristics of patients with PVT and without PVT are summarized in Table 1.

### 3.5. Follow-Up

During a mean follow-up of 803 days, none of the patients experienced portal hypertension-related gastrointestinal bleeding, intestinal infarction, or long-term complications such as esophageal varices, portal biliopathy, splenomegaly, or further thrombosis elsewhere.

## 4. Discussion

COVID-19 has been associated with an increase in thromboembolic events, occurring in up to 20% of hospitalized patients, most commonly in the form of pulmonary embolism (with a prevalence of 17% described in hospitalized patients) and with some type of coagulopathy, documented in up to 50% of patients with severe manifestations [4,11,12]. In the case of PVT, its epidemiology is uncertain, since a few case reports and case series reported PVT to be associated with COVID-19.

In our study, the 6-month cumulative incidence of PVT is 1/1000 patients over 6 months. It was assumed that this incidence will not increase further more than 6 months after the acute event of the initial viral infection, supported by the short time from the positive RT-PCR to diagnosis—a maximum of 21 days—in the three patients. A large epidemiologic study showed that the incidence of PVT was 3.78 and 1.73 per 100,000 inhabitants in males and females, respectively [13], which is lower than the pooled incidence of PVT in the COVID-19 patients showed in our study. In a study of almost 24,000 autopsies in Sweden, the PVT prevalence at autopsy, an expression of lifetime cumulative incidence, was 1% and of these, 28% had cirrhosis [14]. In other studies, the prevalence has been established at 0.7 and 3.7 per 100,000 habitants [15]. Interestingly, however, in a meta-analysis on histopathological reports from deceased COVID-19 patients undergoing autopsy or liver biopsy, hepatic vascular thrombosis was one the main histological findings, with a prevalence of 29.4% in spite of a low prevalence of known chronic liver disease. Therefore, microthrombosis phenomena and thrombosis of sinusoidal vessels could be more frequent than the findings of partial or complete thrombosis of larger caliber vessels that can be seen in abdominal imaging tests [7].

In our study, 5% of patients developed PVT. However, the real percentage could be above 5%, taking into account that there may be patients who did not have portal vein thrombosis at the time of the abdominal CT scan and developed it later with an asymptomatic course. Meanwhile, a meta-analysis demonstrated that 0.6% (95% CI = 0.3% to 1.1%) of COVID-19 patients developed thrombosis of the splenoportal axis with significant heterogeneity (I^2^ = 77.7%; *p* < 0.0001) [16]. However, these data come from case series or observational studies whose main objective was not to estimate the presence of portal thrombosis, but presented this as an incidental finding. Since most patients do not undergo an abdominal imaging test, it is difficult to know the true prevalence of portal thrombosis, since many cases will be asymptomatic, and this could contribute to its underestimation.

The mechanisms that give rise to the strong relationship between SARS-CoV-2 infection and venous and arterial thromboembolism are not clear yet, and it has been hypothesized that it could be different at different sites of the venous vasculature. The main underlying mechanisms include endothelial injury, increased cellular or plasma components, and an excessive inflammatory response that activates the coagulation cascade, leading to increased blood viscosity and resulting in in situ thrombosis in the capillaries and microvessels [17,18,19].

The prothrombotic changes from COVID-19 persist over time-elevated factor VIII, and plasminogen activator inhibitor type 1 levels have been observed at four months post-recovery [20]. However, according to the meta-analysis by Kheyrandish et al. [21] from case report studies, the number of days from the start of COVID-19 symptoms until a PVT diagnosis was 8.3 days. In our case, PVT appeared slightly later, between one and three weeks from the onset of symptoms.

Regarding the factors related to the appearance of thrombosis, the following have been described: male sex, severe disease, pulmonary embolism, higher levels of D-dimer, lactate dehydrogenase (LDH), and white blood cells (WBCs). However, traditional venous thromboembolism risk factors (i.e., history of cancer, previous venous thromboembolism events, obesity) have not been found to be associated with venous thromboembolism in COVID-19 [12,22], and no differences were found in our study, probably due to the small sample size.

PVT is a thromboembolic complication that usually occurs along with an underlying pathology such as cirrhosis, pancreatitis, intra-abdominal infections, inflammatory diseases, and lymphoproliferative or myeloproliferative syndromes. In the latter, JAK2-V617F mutation plays a critical role in the secretion of inflammatory cytokines, which significantly contributes to pathological thrombus formation [23]. For this reason, a thrombophilia study was carried out (including testing for the factor-V-Leiden mutation, the G20210A prothrombin gene mutation, the JAK 2 mutation, protein C and S deficiency, and antithrombin deficiency), which was negative in all patients. In our study, two out of three patients presented none of these pathologies. In the case of the patient with cirrhosis, an abdominal Doppler ultrasound had been performed one month before which ruled out PVT, so COVID-19 may have played a relevant role.

Rare causes of portal hypertension or portal vein thrombosis have been described, such as gastric duplication cysts. The mechanism by which this occurs is due to compression of the splenic vein, which caused portal vein dilatation and the occurrence of left-sided portal hypertension [24]. In our study, the CT images did not reveal data suggestive of abdominal malformations.

The most frequent clinical presentations are abdominal pain (61%) and ascites (83%), while about 20% of the patients are asymptomatic [18]. Data from PVT after COVID-19 indicates that abdominal pain was the most prevalent presentation (66%) [16,21,25,26], as it was in our study (67.7%). There was a trend, although not significant, in patients with PVT (100% vs. 49.1%, *p* = 0.131) to be mostly male compared to non-PVT patients and to be younger (mean age 48.33 ± 14.8 vs. 67 ± 16.19 years, *p* = 0.056). A systematic review of PVT in patients with COVID-19 also observed that males were the most frequent gender (males comprised 54.83–62.1% of subjects) and the age at diagnosis was lower when compared to PVT due to cirrhosis (mean age ranging between 45.1 ± 19.68 and 57.2 ± 11 years) [26].

The use of LMWH in the first 24 h after admission and maintained for a week has been shown to reduce the appearance of thromboembolic phenomena and also mortality at 28 days [27]. However, these data have not been evaluated for the incidence of portal vein thrombosis. In our study, all patients who received LMWH continued receiving the treatment for at least 2 weeks. There were no differences in PVT occurrence with LMWH use. In addition, in our study, up to 66.7% of patients with PVT received LMWH at a therapeutic dose. Despite the use of LMWH, PVT occurred. This could be explained by the fact that the inflammatory and prothrombotic changes produced by COVID-19 began before the patients were admitted and therefore received LMWH.

Preoperative radiological criteria predictive of thrombosis include the splenic vein diameter and splenic volume [28]. The only patient who had a Doppler ultrasound prior to thrombosis was the patient with cirrhosis, whose portal vein diameter was 11 mm (slightly lower than the cut-off point of 12.5 mm, which confers a higher risk of thrombosis to patients with cirrhosis) [29]. Liver stiffness in the patient with cirrhosis was 24.5 kPa, although it was already previously increased due to his liver disease. In the other two patients, it was normal. If PVT persists over time, it may lead to an increase in liver stiffness when hepatic fibrosis occurs [30]. In our case, liver stiffness was normal in the two patients without cirrhosis, since it was an acute thrombosis and did not persist over time. On the other hand, PVT may cause an increase in splenic elastography even in the absence of cirrhosis, while liver stiffness remains normal, and this can be useful to predict the existence of non-cirrhotic portal hypertension [31].

The prognosis of patients with PVT was good and none developed complications during follow-up. As has been described in other studies, PVT did not impair the prognosis of SARS-CoV-2 infection, as no patients died. However, different studies have associated severe COVID-19 with the presence of thrombosis in different territories, findings that did not show a relationship in our study [10,32,33].

The findings of our study also support the increasingly recognized notion in the literature of recent years of an association of viral infection with vein thrombosis, defined as “virus-associated thrombophlebitis”, that could have its own characteristics and different thrombotic properties, even among different types of viruses [34,35]. These considerations suggest that it would be meaningful to distinguish, in future research, three groups of thrombophlebitis defined by major microbiological agents, namely bacteria-associated thrombophlebitis, mycosis-associated thrombophlebitis, and virus-associated thrombophlebitis.

Our study has several limitations, and the results should be interpreted with caution. First, this was a retrospective study. Second, the small number of patients with PVT leads to a low statistical power. A major implication of this is that it reduces the likelihood of detecting statistically significant differences that may exist, and it could explain non-significant research findings in most comparisons. Therefore, the low frequency of cases with PVT means that larger sample sizes are required to confirm the results, and further prospective studies are needed to aid interpretation.

## 5. Conclusions

In conclusion, while major thrombotic events are well-recognized complications of COVID-19, few cases of PVT have been reported in the literature. The present retrospective cohort study is one of the few studies that has evaluated the 6-month cumulative incidence of PVT in a cohort of patients with COVID-19 and assessed their clinical characteristics. Its findings should be confirmed and integrated in studies with alternative designs suitable to investigate rare diseases, including case–control studies, cohort studies, or matched cohort studies in order to achieve a better understanding of the relationship between PVT and COVID-19.

## Figures and Tables

**Table 1:** Patient and laboratory characteristics of the studied groups.

	Non-PVT (*n* = 57)	PVT (*n* = 3)	*p*
Male sex—*n* (%)	28 (49.1%)	3 (100%)	0.131
Age	67 ± 16.19	48.33 ± 14.8	0.056
Smokers—*n* (%)	4 (7%)	0 (0%)	1
Hypertension—*n* (%)	33 (57.9%)	2 (66.7%)	1
Diabetes—*n* (%)	17 (29.8%)	1 (33.3%)	1
Hypercholesterolemia—*n* (%)	21 (36.5%)	0 (0%)	0.545
Thrombophilia—*n* (%)	0 (0%)	0 (0%)	
Liver disease without cirrhosis—*n* (%)	7 (12.3%)	0 (0%)	0.354
Cirrhosis—*n* (%)	1 (1.8%)	1 (33.3%)	0.049
Cardiovascular diseases—*n* (%)	16 (28.1%)	1 (33.3%)	1
Anticoagulant treatment—*n* (%)	14 (24.6%)	0 (0%)	1
Antiplatelet treatment—*n* (%)	10 (17.5%)	0 (0%)	1
BRESCIA scale—*n* (%)			
0	32 (56.1%)	1 (33.3%)	0.583
1–3	23 (40.4%)	2 (67.7%)	0.565
≥4	2 (3.5%)	0 (0%)	1
Lung consolidation in chest rx—*n* (%)			
<25%	19 (33.3%)	1 (33.3%)	1
25–50%	24 (42.1%)	2 (66.7%)	0.574
>50%	14 (24.6%)	0 (0%)	1
Hospital admission—*n* (%)	51 (89.5%)	3 (100%)	1
Intensive care unit admission—*n* (%)	9 (15.8%)	0 (0%)	1
Prophylactic LMWH—*n* (%)	28 (50%)	1 (33.3%)	1
Anticoagulant LMWH—*n* (%)	14 (25%)	2 (66.7%)	0.176
Pulmonary embolism—*n* (%)	5 (8.8%)	1 (33.3%)	0.279
Deep vein thrombosis—*n* (%)	3 (5.4%)	0 (0%)	1
Death—*n* (%)	10 (17.9%)	0 (0%)	1
C-reactive protein (mg/L)	131 (31.9–250)	139.3 (94.2–139.3)	0.631
Serum ferritin (ng/mL)	1130.3 ± 1232.9	548.7 ± 326.8	0.432
LDH (UI/L)	363.8 ± 191.8	325.6 ± 154.7	0.738
CK (U/L)	318.7 ± 713.3	188.5 ± 156.3	0.800
ALT (U/L)	39.34 ± 38.4	56 ± 9.5	0.460
AST (U/L)	44.6 ± 36.4	74.17 ± 84.9	0.608
Bilirubin (mg/dL)	1.1 ± 2.1	4.4 ± 6.5	0.468
Platelets (U/×109/L)	239.3 ± 112.7	193.7 ± 140	0.502
Neutrophil-to-lymphocyte ratio	7.5 ± 6.7	13.2 ± 14.9	0.252
Prothrombin time	49.7 ± 60.9	21.2 ± 11.3	0.505
Activated partial thromboplastin time (s)	36 (31.7–40.1)	37 ± 15.8	0.807
Fibrinogen (mg/dL)	523.6 ± 203.9	423 ± 239	0.499
D-dimer (ng/mL)	4.3 ± 11	1653 ± 1412.1	0.18

PVT: Portal vein thrombosis, LMWH: low-molecular-weight heparin.

## Data Availability

Data are available upon reasonable request to the corresponding author.
